# Microtubule Cytoskeleton Dysfunction in Chronic Pain: Mechanisms of Transport Failure and Emerging Therapeutic Targets

**DOI:** 10.3390/ijms27188135

**Published:** 2026-09-12

**Authors:** Zheng Li, Jie Liu, Tiantian Chu, Feng Gao

**Affiliations:** Department of Anesthesiology and Pain Medicine, Hubei Key Laboratory of Geriatric Anesthesia and Perioperative Brain Health, Wuhan Clinical Research Center for Geriatric Anesthesia, Tongji Hospital, Tongji Medical College, Huazhong University of Science and Technology, Wuhan 430030, China

**Keywords:** chronic pain, microtubule, transport

## Abstract

Chronic pain, a devastatingly prevalent condition, is increasingly understood not merely as a disorder of neuronal signaling but as a failure of the neuronal infrastructure itself. Central to this paradigm is the microtubule (MT) cytoskeleton, which functions not as a passive scaffold but as a dynamic regulatory hub and the primary railway for intracellular transport. This review provides a comprehensive mechanistic analysis of how the dysregulation of microtubule dynamics drives the initiation and maintenance of chronic pain. We dissect the change in microtubule dynamics across diverse pain etiologies, summarizing how diverse insults lead to either pathological hyper-stabilization or catastrophic disassembly of the microtubule network. Finally, we explore emerging therapeutic strategies that move beyond broad-spectrum microtubule modulators to target specific regulatory proteins such as Histone deacetylase 6 (HDAC6), Collapsin Response Mediator Protein 2 (CRMP2), Heat Shock Protein 27 (HSP27), and motor proteins (kinesins and dyneins). We propose that restoring cytoskeletal homeostasis, by which we recalibrate the tubulin code or rescue motor-driven transport, represents a paradigm-shifting approach to pain management. By moving the therapeutic focus from blocking electrical signals to repairing the structural and logistical integrity of the nociceptive neuron, these strategies hold the potential to modify the underlying disease process, offering a new frontier for analgesic development.

## 1. Introduction

Chronic pain remains one of the most prevalent and debilitating health conditions worldwide, affecting more than 10% of the global population. Among these, neuropathic pain constitutes a significant and particularly challenging subset [1,2]. Chronic pain often leads to sleep disturbances, anxiety, depression, and cognitive decline [3]. Thus, it drastically impairs quality of life and imposes an immense burden on individuals and healthcare systems. Despite the extensive armamentarium of analgesics currently available, such as opioids, gabapentinoids, and antidepressants, a substantial proportion of patients continue to experience inadequate pain relief [4]. Moreover, the long-term use of these medications is frequently limited by severe side effects, such as tolerance, addiction, and systemic toxicity. Consequently, there is an urgent and unmet clinical need to identify novel therapeutic targets and pathophysiological mechanisms that transcend the traditional focus on ion channels, neurotransmitter receptors, oxidative stress, and programmed cell death [5,6,7].

Emerging evidence suggests that the neuronal cytoskeleton, specifically microtubules, serves as a critical yet underappreciated player in the pathophysiology of pain [8,9]. Microtubules are dynamic polymers composed of α- and β-tubulin heterodimers that provide essential structural support and act as the primary railways for intracellular transport in neurons [10]. Intact microtubule tracks are essential for the anterograde and retrograde trafficking of organelles, proteins, and signaling molecules [11]. This transport system, powered by motor proteins (kinesins and dyneins), is finely regulated by the diverse array of microtubule post-translational modifications (PTMs) such as acetylation, detyrosination, and polyglutamylation [12,13]. Recent research has begun to uncover that disruptions in microtubule dynamics and stability are central to various pain etiologies [8,9,14,15]. For instance, chemotherapeutic agents like paclitaxel induce neuropathic pain by hyper-stabilizing microtubules and disrupting axonal transport [14], while novel findings indicate that modulating microtubule acetylation via Histone deacetylase 6 (HDAC6) inhibition can alleviate sensory hypersensitivity [15]. These insights position the microtubule cytoskeleton not merely as a structural scaffold but as a dynamic signaling hub actively involved in nociception.

In this review, we aim to synthesize the current understanding of the role of microtubules in pain processing. We will discuss how distinct pathological insults lead to excessive microtubule stabilization or depolymerization and how these structural changes impair axonal transport to drive pain progress. Furthermore, we will explore the therapeutic potential of targeting the tubulin cytoskeleton, tubulin code and microtubule-associated enzymes, offering a perspective on how restoring cytoskeletal homeostasis could represent a paradigm shift in the management of chronic pain.

## 2. Architecture of the Neuronal Microtubule Cytoskeleton

Microtubules (MTs) are dynamic, polar cytoskeletal polymers that serve as essential scaffolds and intracellular highways in eukaryotic cells [1]. They are composed of α/β-tubulin heterodimers that assemble head-to-tail to form protofilaments. This assembly imparts a distinct structural polarity [16], distinguishing a rapidly growing plus-end (+) from a relatively stable minus-end (−) anchored at the centrosome or other microtubule-organizing centers (MTOCs) [3,5]. The polymerization process is intrinsically dynamic, driven by the hydrolysis of Guanosine Triphosphate (GTP) bound to β-tubulin; the loss of the GTP cap triggers stochastic switching between growth and shrinkage, a phenomenon known as microtubule dynamic equilibrium [16,17].

The architecture of the microtubule cytoskeleton is not merely defined by its polymeric structure but is fine-tuned by a sophisticated regulatory network comprising post-translational modifications (PTMs) and microtubule-associated proteins (MAPs) [9,10,11,12,13]. This regulatory layer is often referred to as the “tubulin code” [10]. The majority of these modifications occur on the C-terminal tails of tubulins, which project outward from the microtubule surface. These modifications include acetylation, detyrosination/retyrosination, polyglutamylation, and phosphorylation [10]. Additionally, by altering the surface chemistry of the microtubule, these PTMs act as specific “signaling languages” that recruit distinct sets of MAPs and motor proteins. This process modulates the stability, mechanics, and transport capacity of the cytoskeleton without changing the underlying polymer structure. Meanwhile, the behavior of microtubules is also tightly controlled by a diverse array of MAPs that stabilize, destabilize, or remodel the cytoskeleton [9,10,12,18]. Stabilizing proteins, such as Tau, MAP2, and MAP6 (STOP), bind along the lattice to reduce catastrophe frequency and promote bundling, thereby increasing structural resilience. Conversely, destabilizing factors like Op18/Stathmin sequester tubulin dimers or promote catastrophe to facilitate rapid remodeling [18]. Furthermore, severing enzymes, including katanin and spastin, use Adenosine Triphosphate (ATP) hydrolysis to cut microtubules internally, generating new plus ends that are essential for branching, transport, and the rapid reorganization of the network in response to cellular signals. This complex interplay between dynamic instability, PTMs, and MAP activity allows cells to precisely calibrate their microtubule cytoskeleton to meet physiological demands.

## 3. Function of the Neuronal Microtubule Cytoskeleton

**Figure 1:**
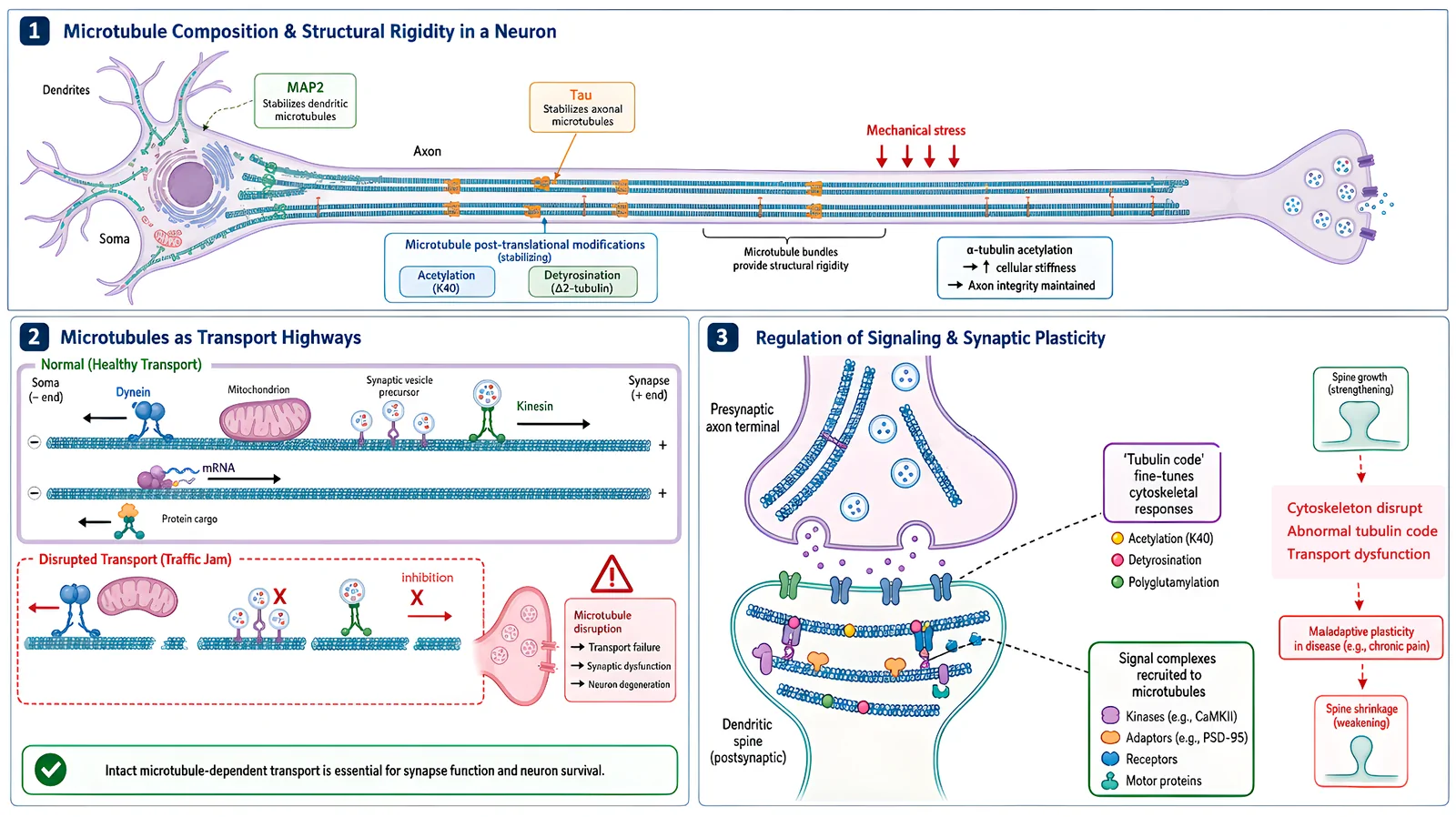
Function of the Neuronal Microtubule Cytoskeleton. MAP2, microtubule-associated protein 2; PSD-95, Postsynaptic Density Protein 95. (The Figure 1 was created with www.figurelabs.ai. Retrieved from https://chat.figurelabs.ai/verify/FL-PUB-20260820-94OK25 on 20 August 2026. The generated content may be used for academic publication, and the authors have obtained the required license for this use. The generated image was subsequently modified, edited, and refined using Adobe Photoshop CS5).

Microtubules are fundamental to the development and maturation of the nervous system, playing a pivotal role in orchestrating the complex processes of neuronal migration, polarization, and differentiation [19] (Figure 1). During early development, dynamic microtubules drive the nucleokinesis essential for neuronal migration, where the coordinated extension and retraction of leading processes guide neurons to their correct laminar positions [20]. In the mature axon, microtubules acquire a uniform orientation with their plus-ends (+) directed distally, a configuration that is pivotal for defining the axon as the sole output domain of the neuron [19]. Meanwhile, microtubules continuously undergo depolymerization, aggregation, and even nucleation in response to neural synapse signals [21,22]. Beyond their role in shaping morphology, microtubules provide the mechanical rigidity required to sustain the integrity of long axonal processes [23,24,25,26]. Given that axons can extend for over a meter in humans, they must resist physical stress and prevent breakage. This mechanical resilience is achieved through the bundling of microtubules into parallel arrays by MAPs such as Tau and MAP2 [23,24,26]. Furthermore, specific microtubule subsets acquire stability through post-translational modifications like acetylation and detyrosination [10,25,27], rendering them resistant to cold and chemical depolymerization. Notably, α-tubulin acetylation modulates cellular stiffness, thereby directly setting the mechanical threshold required for touch sensation without regulating other mechanisms [28]. These stable tracks serve as a persistent scaffold, maintaining the structural health of the neuron. These structural foundations not only define neuronal morphology but also underpin the neuron’s ability to form precise synaptic connections and integrate into complex neural circuits.

Perhaps the most critical function of microtubules in neurons is their role as the primary highways for axonal transport [29,30]. Due to the extreme distance between the cell body (site of synthesis) and synaptic terminals, neurons rely on efficient intracellular trafficking to deliver organelles, vesicles, proteins, and mRNAs. Microtubules serve as the tracks for motor proteins: kinesins generally drive anterograde transport toward the microtubule plus-end (synaptic terminals), while dyneins mediate retrograde transport back to the soma [31]. This system is essential for the distribution of mitochondria, which supply ATP to energy-demanding regions like the node of Ranvier and active synapses, as well as for the replenishment of synaptic vesicles and the removal of degraded proteins. Notably, disruption of this transport network due to microtubule instability (fragmentation or hyper-stability) can lead to “traffic jams”, resulting in synaptic dysfunction, neuronal hyperexcitability, and ultimately neurodegeneration [29,30,32,33].

Beyond their structural and transport roles, microtubules act as active regulators of local signaling and synaptic plasticity [30,34]. They serve as scaffolds for signaling complexes, positioning receptors and kinases at specific subcellular locations to modulate signal transduction cascades. For instance, microtubules regulate endoplasmic reticulum–plasma membrane (ER-PM) contact, which is crucial for maintaining intracellular calcium homeostasis and lipid signaling [35,36]. The “tubulin code”, comprising diverse post-translational modifications, acts as a signaling language that recruits specific MAPs and motors, thereby fine-tuning the cytoskeletal response to extracellular cues [10]. This capacity for local regulation allows microtubules to rapidly reorganize in response to neuronal activity, facilitating structural changes at the synapse that underlie learning and memory [37]. Conversely, dysregulation of this dynamic plasticity contributes to maladaptive changes in pathological states such as chronic pain, where aberrant microtubule signaling drives persistent neuronal sensitization [38,39].

## 4. The Dysregulation of the Neuronal Microtubule Cytoskeleton in Chronic Pain Pathology

While the microtubule cytoskeleton is indispensable for maintaining neuronal homeostasis, the dysregulation of microtubule dynamics has been implicated as a convergent mechanism contributing to the initiation and maintenance of chronic pain. Among the various pain etiologies, this causal relationship is most clearly established in chemotherapy-induced peripheral neuropathy (CIPN), where microtubule-targeting agents (e.g., paclitaxel, vincristine) act as the primary pathological insult, and where microtubule disruption has been directly linked to neuronal dysfunction. Evidence for microtubule involvement has also been documented in other chronic pain states, including diabetic peripheral neuropathy, inflammatory pain, and nerve injury-induced neuropathic pain. However, although experimental findings consistently implicate microtubule dysregulation across diverse pain models, further investigation is required to definitively establish the causal relationship between microtubule dysfunction and chronic pain in each specific context and to distinguish whether microtubule changes represent a primary mechanism or a downstream consequence of other pathological events such as inflammation. In the following sections, we summarize the current evidence for microtubule dysregulation in each of the major chronic pain etiologies.

### 4.1. Chemotherapy-Induced Peripheral Neuropathy (CIPN)

**Figure 2:**
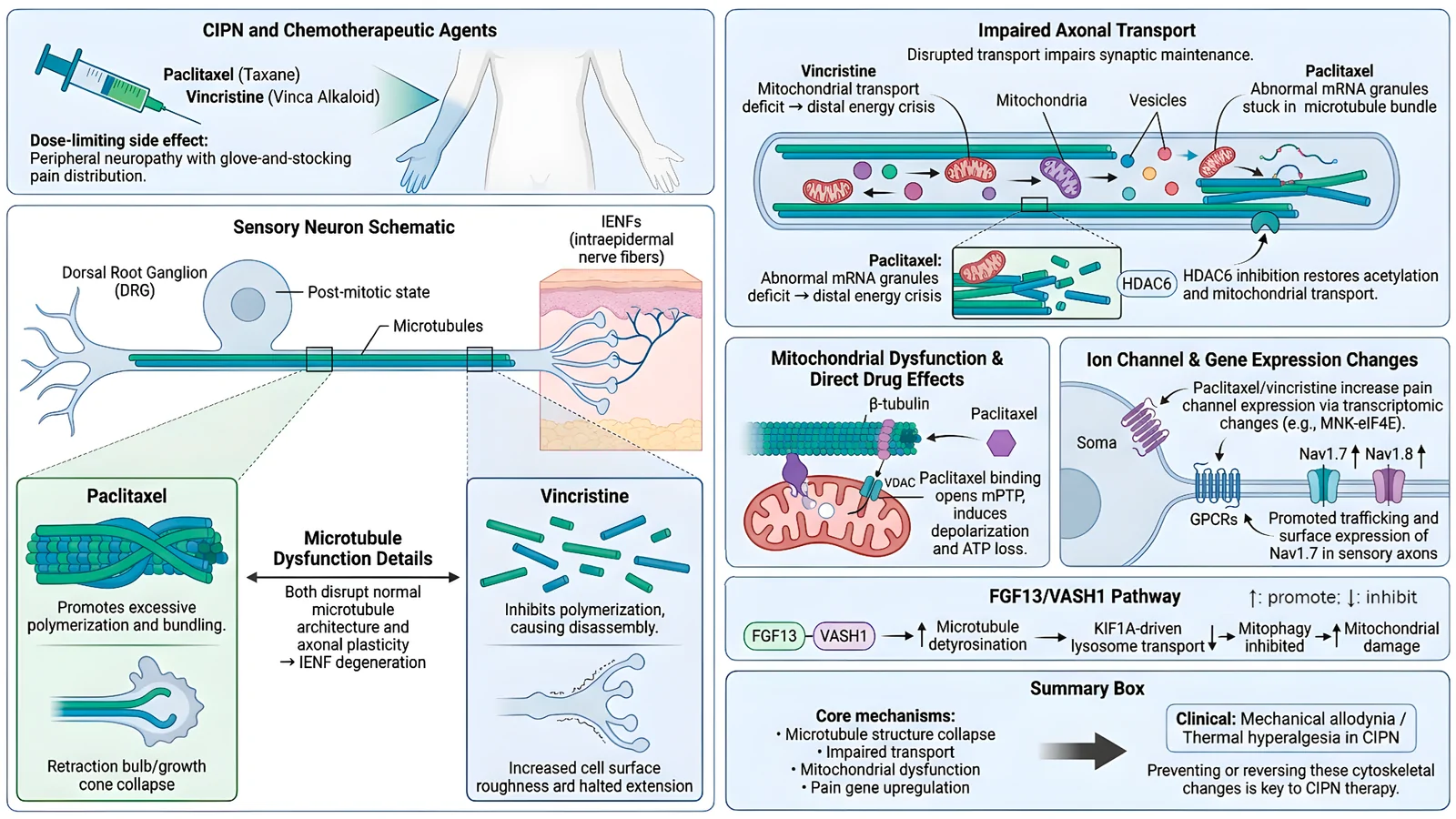
The change and influence of microtubule cytoskeleton in CIPN. CIPN, chemotherapy-induced peripheral neuropathy; IENFs, intraepidermal nerve fibers; DRG, dorsal root ganglion; HDAC6, Histone deacetylase 6; mPTP, mitochondrial permeability transition pore; ATP, Adenosine Triphosphate; MNK, Mitogen-Activated Protein Kinase-Interacting Kinase; eIF4E, Eukaryotic Translation Initiation Factor 4E; GPCRs, G Protein-Coupled Receptors; FGF13, fibroblast growth factor 13; VASH1, vasohibin 1. (Figure 2 was created with www.figurelabs.ai. Retrieved from https://chat.figurelabs.ai/verify/FL-PUB-20260820-QDGLYU on 20 August 2026. The generated content may be used for academic publication, and the authors have obtained the required license for this use. The generated image was subsequently modified, edited, and refined using Adobe Photoshop CS5).

CIPN is a dose-limiting side effect of several potent anti-cancer drugs, particularly taxanes (e.g., paclitaxel) and vinca alkaloids (e.g., vincristine) [40]. Clinically, patients present with a “glove and stocking” distribution of pain, characterized by mechanical allodynia and thermal hyperalgesia. This pathology arises primarily because these agents target rapidly dividing cells but also inadvertently affect the highly dynamic microtubule network of post-mitotic sensory neurons (Figure 2).

Paclitaxel promotes excessive tubulin polymerization, leading to the formation of rigid, hyper-stable bundles, while vincristine inhibits polymerization, causing microtubule disassembly [41]. Both extremes destabilize the neuronal architecture. For instance, vincristine treatment disrupts the packing density of microtubules, increasing cell surface roughness and halting neurite extension [42]. Similarly, paclitaxel promotes the formation of retraction bulb-like structures at distal tips, indicative of growth cone collapse [14]. This loss of plasticity in remodeling axonal terminals triggers the degeneration of distal intraepidermal nerve fibers (IENFs) [14], a structural correlate of the altered pain perception and hypersensitivity observed in CIPN patients [43].

Disruption of microtubule dynamics by chemotherapeutic agents leads to severe transport deficits. Vincristine, by depolymerizing the microtubule lattice, physically stalls mitochondrial motility, leading to energy deprivation at the distal axon and subsequent neurite degeneration [44,45]. Although paclitaxel creates rigid tracks rather than destroying them, the hyper-stabilization and bundling of microtubules alter their polarity and geometric organization [32,33], which similarly impairs the transport of mRNA granules [46]. This deficit in mRNA delivery compromises the local translation capacity required for synaptic maintenance. Thus, the relationship between microtubule stability and transport efficiency is bidirectional and context-dependent: both extremes destabilize the transport machinery, but through distinct mechanisms: paclitaxel by rigidifying the track and altering its surface chemistry, and vincristine by disassembling the track altogether. Notably, pharmacological inhibition of HDAC6 restores microtubule acetylation, which participates in microtubule stability, and rescues mitochondrial transport deficits [45] (see Section 5.2 for detailed discussion of HDAC6), but no strategy was found to inhibit paclitaxel-induced microtubule hyper-stability for chronic pain. These suggest that the microtubule dynamics disruption and surface modifications induced by chemotherapy are critical determinants of transport efficiency.

Mitochondrial integrity is inextricably linked to the microtubule cytoskeleton. Beyond serving as anchors for mitochondrial transport, microtubules interact directly with mitochondrial components. For example, paclitaxel binds to the β-tubulin present in the mitochondrial membrane and Voltage-Dependent Anion Channel (VDAC), opening the mitochondrial permeability transition pore (mPTP) and inducing rapid depolarization [47,48]. This direct chemical interaction, coupled with the structural collapse of the axon, leads to severe mitochondrial phenotypes, including swelling and vacuolization in both myelinated and unmyelinated fibers [49,50]. Consequently, ATP production is compromised, creating an energy crisis in energy-demanding nociceptors, highlighting that cytoskeletal preservation is essential for mitochondrial bioenergetics.

In addition to abnormal axonal transport of mitochondria and axonal cargos induced by microtubule dysfunction, microtubule cytoskeleton can deadly induce transcriptomic changes in pain-related channels [51,52,53,54]. Paclitaxel treatment induced upregulation of G protein-coupled receptors and ion channels in both dorsal root ganglions (DRGs) and spinal dorsal horns [53,54]. Mechanistic studies revealed that the paclitaxel-induced transcriptomic changes were involved in Mitogen-Activated Protein Kinase-Interacting Kinase (MNK)–Eukaryotic Translation Initiation Factor 4E (eIF4E) signaling [51]. Similarly, vincristine treatment upregulated the expression of Nav1.7/Nav1.8 channels in rat dorsal root ganglion (DRG) neurons [55] and spinal cord [56]. Combined with the promoting effect of chemotherapeutic agent treatment on trafficking and surface distribution of Nav1.7 in sensory axons [57,58], the increased number of pain-related ion channels contributes to the development of CIPN [41,55,56,57,58].

Furthermore, fibroblast growth factor 13 (FGF13) has been shown to physically interact with vasohibin 1 (VASH1), regulating VASH1 binding to microtubules and promoting detyrosination. This finding suggests that FGF13 is involved in microtubule modification within this specific context. The resulting detyrosination prevents kinesin-3 (KIF1A)-driven lysosomal trafficking, inhibits mitophagy, and ultimately contributes to paclitaxel-induced mitochondrial damage and CIPN [59]. However, it remains to be validated whether this specific relationship between FGF13 and microtubule regulation represents a general mechanism underlying CIPN pathology or is specific to this experimental paradigm.

### 4.2. Diabetic Peripheral Neuropathy (DPN)

DPN is a common complication of diabetes mellitus, driven by chronic metabolic stressors including hyperglycemia, oxidative stress, and the formation of advanced glycation end-products (AGEs) [60]. It manifests as a symmetrical, length-dependent sensory loss and pain, often described as burning or lancinating. The metabolic environment slowly erodes the cytoskeletal foundation of sensory neurons.

A previous study found that microtubule numbers and densities were similarly reduced in large myelinated axons and microtubule numbers reduced in small myelinated and unmyelinated axons in chronic diabetes. And axonal atrophy was observed in unmyelinated fibers. Meanwhile, the decreased mRNA expression of cytoskeletal proteins found in sensory neurons accompanies a reduction in their incorporation into distal axons [61]. These changes imply that there is a direct link between diabetic pathology in the sensory neuron and alterations of its distal branches from diabetes. Mechanistically, chronic hyperglycemia drives cytoskeletal disruption through several distinct pathways. One major mechanism involves the formation of advanced glycation end-products (AGEs) [62], which covalently modify tubulin and neurofilaments, inhibiting GTP-dependent microtubule polymerization and stiffening the cytoskeletal architecture [63,64]. Additionally, the binding of AGEs to their receptor (RAGE) on neurons interacts with the cytoskeletal protein DIAPH1, further disrupting microtubule organization [65]. Parallel to this, glycogen synthase kinase-3β (GSK-3β), a key factor elevated in insulin deficiency and resistance, serves as a critical coordinator of neuronal maintenance [66]. In neurons, GSK-3β regulates the cytoskeleton by phosphorylating microtubule-binding proteins; its misregulation has been shown to induce pathological axonal swellings characterized by disorganized, curled microtubule bundles [67]. These findings imply that while microtubule disruption is a common endpoint in diabetes, it arises from multiple upstream insults involving different structural appearances.

The microtubule injury induced by diabetes is not merely a structural anomaly; it serves as the critical upstream driver of the distal axon degeneration that characterizes diabetic neuropathic pain. The transition from cytoskeletal damage to the pain phenotype is mainly mediated through specific failures in axonal transport and energy homeostasis [45,63,68]. A primary mechanism linking microtubule destabilization to pain is the deficit in mitochondrial transport, which induces ATP energy deprivation [45,63,68]. Microtubule disorganization also impedes the retrograde transport of neurotrophic factors, such as Nerve Growth Factor (NGF), from the periphery to the DRG cell body. The deprivation of trophic support triggers maladaptive transcriptional changes within the neuron, further leading to compromised axonal functions and eventual atrophy and loss [69]. Although each of these mechanisms has been individually documented in experimental systems, the extent to which they converge into a single causal cascade in diabetic neuropathy remains incompletely defined. For example, in addition to the influence of microtubule disruption, mitochondrial axonal transport is inhibited by local rising Ca^2+^ levels mediated by transient receptor potential cation channel subfamily V member 4 (TRPV4) [70,71]. Ca^2+^ fluctuations gravely alter mitochondrial function and movement [71,72], and rising Ca^2+^ levels can inhibit mitochondrial axonal transport directly through Ca^2+^ association with the Miro, a mitochondrial surfactant protein involved in docking with the motor domain of kinesin-1 [73]. Thus, more research needs to be carried out to explore the link between cytoskeletal damage and DPN, involved in cargo transport and energy supply.

### 4.3. Inflammatory Pain

Inflammatory pain is caused by tissue injury or infection, leading to the release of inflammatory mediators (e.g., NGF, prostaglandins, cytokines) that sensitize peripheral nociceptors (peripheral sensitization) [74]. Unlike the neurodegeneration seen in CIPN or DPN, the primary issue in inflammatory pain is maladaptive plasticity—changes in the cytoskeleton that facilitate excitability.

A recent study found that the role of FGF13 in neuronal excitability of DRG was linked to its modulation of voltage-gated Na^+^ channels mediated by microtubules in complete Freund’s adjuvant (CFA)-induced chronic inflammatory pain [75]. Specifically, FGF13 could interact with microtubules and stabilize microtubules in DRG neurons and increase the expression and function of Na^+^ channel proteins in the surface membrane. These results suggest that the regulation of the increased stability of microtubules may be an important mechanism for translocation of Na^+^ channels to the cell surface membrane in DRG neurons and inflammatory pain [75]. Additionally, the increased stability of microtubules can facilitate trafficking and surface delivery of N-methyl-D-aspartate receptor (NMDAR) [76], Nav1.7 channels [77], and vanilloid receptor subtype 1 (VR1) [78], which induce mechanical hypersensitivity and hyperalgesia in inflammatory pain. In addition to the influence of the microtubule cytoskeleton, these microtubule-dependent transport of receptors and channels are also involved in translocation protein activation, such as the kinesin family, including KIF11 [78], KIF17 [79,80,81], and the stearated Ht31 peptide [76].

Moreover, microtubules can also link locally generated signals in the form of the nascent synthesis of a transcription factor within the peripheral axons of sensory neurons to remote molecular effects within the cell bodies that manifest as altered mechanical hypersensitivity and hyperalgesic priming. For example, inflammatory factor IL-6 could induce a rapid increase in cAMP response element-binding protein (CREB) expression in DRG neurons, and microtubule polymerization facilitates retrograde transport of CREB, in return, leading to nuclear Brain-Derived Neurotrophic Factor (BDNF) expression [82], which initiates and maintains a chronic inflammatory pain [83].

### 4.4. Neuropathic Pain

Neuropathic pain, a major public health problem worldwide, affects the quality of life of 6.9–10% of the general population [84]. Neuropathic pain is characterized by spontaneous ongoing or evoked sensory stimuli (hyperalgesia and allodynia). And it is mainly observed in peripheral nerve lesions (peripheral nerve injury, trigeminal neuralgia) or central nerve lesions (spinal cord injury (SCI)) [85]. However, few studies explored the role of microtubule dynamics in neuropathic pain.

Increased polymerization of microtubules in the DRG neurons was found to be increased in spared nerve injury (SNI)-induced neuropathic pain [86,87]. Consistent with this, pharmacological depolymerization of microtubules by colchicine partially attenuates mechanical and thermal allodynia in the chronic compression of the DRG (CCD) model, an effect associated with reduced TRPV4 expression and decreased substance P (SP) release [88,89], suggesting that enhanced microtubule stability may contribute to neuropathic hypersensitivity, at least in part, by facilitating the trafficking and functional upregulation of pronociceptive channels. In addition, the kinesin motor protein KIF17, which mediates anterograde transport of NMADR2B, was found to be overexpressed in the sciatic nerve, DRG, and spinal cord of nerve-injured rats, and its inhibition attenuated pain hypersensitivity, implicating microtubule-dependent cargo delivery in the maintenance of neuropathic pain [90].

Notably, polymerizations of microtubules were found in both neuropathic and inflammatory pain; certain molecular mechanisms appear to be shared between neuropathic and inflammatory pain, although their pathological contexts differ. A representative example is Nogo-A, a modulator of microtubule assembly. In both SNI-induced neuropathic pain and CFA-induced inflammatory pain, Nogo-A promotes microtubule polymerization and α-tubulin acetylation, thereby enhancing the membrane localization of TRPV1 in DRG neurons and contributing to mechanical and heat hypersensitivity [86,87]. However, while Nogo-A-mediated microtubule remodeling in inflammatory pain likely reflects a form of maladaptive but reversible plasticity, its contribution in neuropathic pain is superimposed on a background of structural nerve injury and may intersect with degenerative signaling pathways. Given that only a limited number of studies have directly compared microtubule dynamics across pain etiologies, the same molecular cascade that produces distinct functional outcomes in these two contexts remains to be determined.

## 5. Therapeutic Implications: Targeting the Cytoskeleton

**Figure 3:**
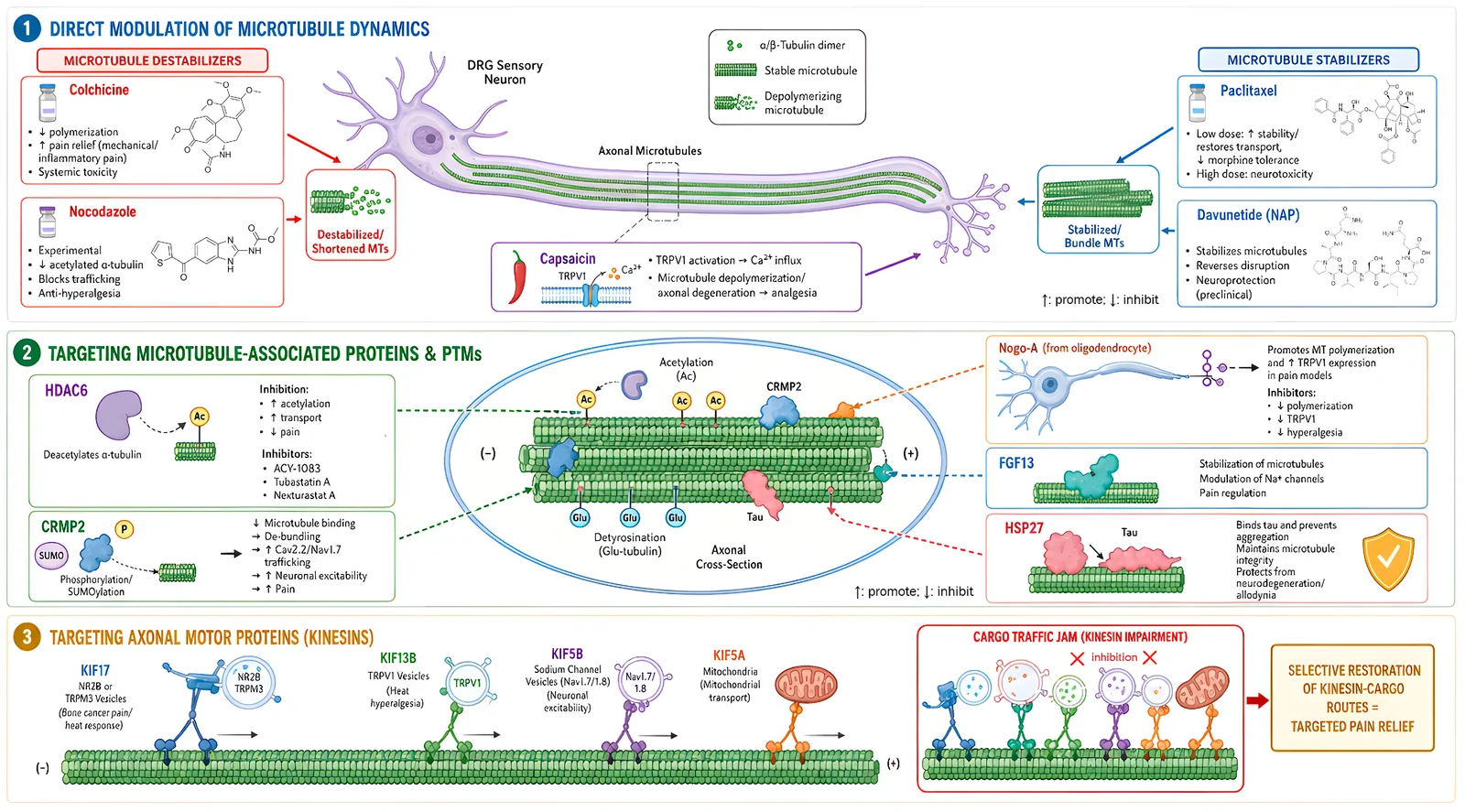
The potential therapeutic agents or mechanisms targeting the microtubule cytoskeleton. DRG, dorsal root ganglion; MT, microtubule; TRPV1, transient receptor potential vanilloid subfamily member 1; NAP, Davunetide; FGF13, fibroblast growth factor 13; HDAC6, Histone deacetylase 6; CRMP2, Collapsin Response Mediator Protein 2; HSP27, Heat Shock Protein 27. (The Figure 3 was created with www.figurelabs.ai. Retrieved from https://chat.figurelabs.ai/verify/FL-PUB-20260724-VXFZ65 on 24 July 2026. The generated content may be used for academic publication, and the authors have obtained the required license for this use. The generated image was subsequently modified, edited, and refined using Adobe Photoshop CS5).

Given the robust causal link established between microtubule dysfunction and the pathogenesis of diverse pain states, targeting the neuronal cytoskeleton emerges as a highly promising and logical therapeutic avenue. The extensive evidence demonstrates that microtubule dynamic dysfunction, post-translational modifications, and the subsequent abnormality of axonal transport drive neuronal hyperexcitability and are involved in chronic pain progression. However, either microtubule hyperpolymerization or microtubule depolymerization (microtubule dynamics dysfunction) can lead to pain processes, so understanding these detailed mechanisms allows for precise therapeutic interventions. Next, the focus of this review now shifts from mechanistic understanding to therapeutic application. Researchers have begun exploring therapeutic strategies aimed at reshaping microtubule dynamics, restoring transport efficiency, or regulating key signaling molecules that drive these cytoskeletal changes (Figure 3).

### 5.1. Direct Modulation of Microtubule Dynamics

The most direct approach involves the pharmacological stabilization or destabilization of microtubules.

#### 5.1.1. Microtubule Destabilizer

Colchicine: Colchicine is FDA-approved for the prophylaxis and treatment of acute gout flares [91]. It acts as a first-line agent, particularly effective when administered early in an attack. Its mechanism involves the inhibition of microtubule polymerization, which disrupts neutrophil chemotaxis, adhesion, and the activation of the NOD-like receptor pyrin domain containing 3 (NLRP3) inflammasome, thereby rapidly reducing the intense inflammation characteristic of gout. While colchicine is highly effective for inflammatory pain associated with the conditions above, its application as a direct analgesic for non-inflammatory or neuropathic pain states remains to be validated. Previous studies found that colchicine could effectively reduce mechanical and thermal hyperalgesia in models like CCD-induced neuropathic pain [88,89]. Although preclinical studies have demonstrated that colchicine’s ability to disrupt microtubule dynamics can modulate the activity of nociceptive ion channels (such as TRPV4) and attenuate hyperalgesia in animal models [88,89], these findings have not yet translated into approved clinical therapies for chronic pain. Currently, as pharmacological tool compounds, the primary value of colchicine lies in demonstrating experimental causality between microtubule dynamics and nociceptive behavior, rather than as realistic candidates for chronic pain therapy. The significant risk of systemic toxicity, particularly neuro-myopathy [92], necessitates extreme caution, limiting its potential for long-term pain management unless targeted delivery systems or safer analogues are developed.

Nocodazole: Intrathecal injection of nocodazole (a microtubule disruptor) could reduce acetylated α-tubulin and significantly attenuate the CFA-induced inflammatory heat hyperalgesia and the mechanical pain in a rat model of SNI [87]. Furthermore, nocodazole could prevent the development of mechanical hypersensitivity and hyperalgesic priming caused by intraplantar injection of Interleukin-6 (IL-6), an inflammatory factor, by disrupting axonal trafficking [82]. However, as pharmacological tool compounds, nocodazole is only applied to research/experimental use to demonstrate experimental causality between microtubule dynamics and nociceptive behavior, rather than as realistic candidates for chronic pain therapy.

#### 5.1.2. Microtubule Stabilizer

Paclitaxel: While high-dose systemic paclitaxel is notorious for causing chemotherapy-induced neuropathy [42], emerging evidence suggests that low-dose or local application can paradoxically alleviate the development of morphine tolerance [93]. By stabilizing the disruption of microtubules by morphine treatment, low-dose paclitaxel could ameliorate synaptic energy deficit via microtubule-based mitochondrial transport. However, its therapeutic window is narrow, as excessive stabilization disrupts dynamic instability essential for plasticity and can lead to axonal stiffening. Although seemingly paradoxical given its well-established role in causing CIPN, the use of low-dose or locally applied paclitaxel to restore microtubule-based transport should be strictly interpreted as an experimental proof-of-concept that microtubule stabilization can be beneficial in a specific context where microtubule is disrupted. Currently, the primary value of paclitaxel lies in demonstrating experimental causality between microtubule dynamics and nociceptive behavior. It does not constitute a rationale for the clinical use of paclitaxel as an analgesic if targeted delivery systems or safer analogues are developed.

Davunetide: Davunetide (NAP, an 8-amino acid peptide derived from activity-dependent neuroprotective protein) is a novel neuroprotective compound with a mechanism of action that appears to involve microtubule stabilization and repair [94]. NAP treatment could reverse axonal transport disruption by colchicine, suggesting drug-dependent protection against axonal transport impairment through stabilization of the neuronal MT network [95]. Currently, NAP is the first neuroprotective peptide and has preclinical evidence for neuroprotective, neurotrophic and cognitive protective properties [96]. It needs to be explored for microtubule disruption by chronic pain.

#### 5.1.3. Capsaicin

Capsaicin treatment can induce analgesia for chronic pain by regulating receptor desensitization of the transient receptor potential vanilloid subfamily member 1 (TRPV1), a key molecule in peripheral sensitization associated with inflammation and injury [97]. In adult rats, the systemic administration of capsaicin at a higher dose (100 mg/kg) induced the degeneration of DRG neurons and unmyelinated axons [98]. This long-term structural ablation is mediated by a TRPV1-dependent influx of Ca^2+^, which triggers localized axonal collapse [99]. Specifically, the mechanisms of microtubule degradation likely involve Ca^2+^-dependent processes. The microtubule cytoskeleton is highly sensitive to Ca^2+^; elevated intracellular Ca^2+^ concentrations enhance depolymerization, presumably by accelerating the hydrolysis of GTP bound to tubulin dimers [8,100]. Consequently, capsaicin-induced TRPV1 activation initiates a Ca^2+^-dependent cascade that disrupts the axonal cytoskeleton by promoting microtubule disassembly [101]. Given that TRPV1 is highly expressed primarily on C and some Aδ nociceptive sensory nerves, particularly those that detect painful or noxious sensations (nociceptors), the degeneration of these unmyelinated axons by capsaicin-induced destabilization of microtubules can reduce the transmission of abnormal pain signals and achieve analgesic effects.

### 5.2. Targeting Microtubule-Associated Proteins and Post-Translational Modifications

To circumvent the toxicity of broad-spectrum microtubule drugs, research has pivoted toward targeting the regulatory proteins that govern microtubule dynamics and transport (Table 1).

HDAC6: HDAC6 is a unique member of the histone deacetylase family (Class IIb). Unlike most other HDACs, which are primarily nuclear and regulate gene expression by modifying histones, it possesses two catalytic deacetylase domains and a C-terminal zinc finger domain (ZnF-UBP) that binds ubiquitin [114,115]. This structural uniqueness allows HDAC6 to function as a critical regulator for not only protein degradation but also the cytoskeleton and cell motility [116]. HDAC6 can remove acetyl groups from lysine 40 (K40) on α-tubulin, leading to the deacetylation of α-tubulin. Deacetylation of tubulin destabilizes the microtubule network and alters its mechanical properties [116]. Pharmacological inhibition of HDAC6 restores microtubule acetylation, which participates in microtubule stability, keeping microtubules in a polymerized state, and rescues mitochondrial transport deficits in vincristine-induced peripheral neuropathies [45]. This participation in microtubule stability suggests that HDAC6 activity can help maintain the polymerized state of the microtubule network, which is essential for its structural role as an axonal transport highway. More importantly, the acetylation status of microtubules serves as a “traffic signal” for motor proteins [117]. By deacetylating microtubules, HDAC6 can impair the transport of vital cargo, such as mitochondria [107] and neurotrophic factors. In chronic pain models, HDAC6 inhibition has been shown to improve mitochondrial transport along microtubules by restoring acetylated microtubules in DRG neurons and prevent mechanical allodynia induced by cisplatin, which has no influence on microtubule stability [107]. In addition to disrupting axonal transport by disrupting microtubule stability and reducing the recruitment of motor proteins, upregulated HDAC6 was found to promote inflammatory cytokine production, leading to inflammatory pathways in chronic pain [102,104,105], making it a promising therapeutic target.

Consequently, significant efforts have been directed toward the development of pharmacological agents capable of selectively inhibiting HDAC6 activity. Currently, selective HDAC6 inhibitors, such as ACY-1083 [107], tubastatin A (TSA) [102], ACY1215 [15,104,105], ACY-738, ACY-257 [118], ITF6464, and ITF6475 [119], have been applied to preclinical chronic pain and have achieved excellent results (Table 2). However, the efficiency of them in clinical therapies needs to be explored.

CRMP2: As a key regulator of microtubule dynamics, CRMP2 can bind to microtubule heterodimers and promotes microtubule assembly and polymerization [121]. By facilitating the formation of microtubule bundles, CRMP2 is essential for neurite outgrowth, axonal guidance, the maintenance of the structural integrity of the neuronal cytoskeleton, and intracellular transport [122]. CRMP2 can be phosphorylated by Cyclin-Dependent Kinase 5 (CDK5) [112] or GSK-3β [123], losing its affinity for cytoskeleton proteins, leading to the inhibition of axonal growth. Neuroprotective effects against SCI, such as microtubule stabilization, reduced inflammation, and suppressed scarring, were also observed by inhibiting CRMP2 phosphorylation [123]. In addition to its fundamental role in stabilizing the cytoskeleton, CRMP2 participates in chronic pain by its ability to regulate the axonal transport and activity of voltage-gated ion channels [112,113]. CRMP2 interacts with and facilitates the forward trafficking of N-type voltage-gated calcium channels (Cav2.2) and sodium channels (Nav1.7) to the membrane. This increases neuronal excitability and neurotransmitter release, leading to central and peripheral sensitization [112,113]. Interestingly, CRMP2 can undergo different types of post-translational modifications to participate in the pain process, such as phosphorylation [112] or SUMOylation [124], making it a promising therapeutic target. Given that CRMP2 and kinesins participate in a broad range of physiological neuronal functions beyond pain signaling, including developmental axon guidance, basal organelle trafficking, and synaptic maintenance, their therapeutic targeting carries a substantial risk of off-target effects. Any clinical translation would require strategies that achieve spatial, temporal, or cargo-specific selectivity.

Nogo-A: Nogo-A is a key inhibitory molecule of axon regeneration in oligodendrocytes and plays diverse roles in other pathological conditions, such as stroke, schizophrenia, and neurodegenerative diseases [125]. Increased Nogo-A can promote microtubule polymerization and contribute to heat hyperalgesia in CFA-induced inflammatory pain via increasing the content of TRPV1 in DRG neurons [86,87]. Inhibiting Nogo-A signaling reduces microtubule polymerization, decreasing the membrane localization of TRPV1 and alleviating thermal hypersensitivity. In a rat model of SNI-induced neuropathic pain, the Nogo-A and acetylated α-tubulin in DRG were also significantly upregulated [87]. This highlights the potential of modulating endogenous regulators of microtubule to nociceptor sensitivity.

FGF13: FGF13, abundantly expressed in DRG neurons, can stabilize microtubules and modulate CFA-induced inflammatory pain and paclitaxel-induced neuropathic pain [59,75]. Conditional knockout of FGF13 can reduce neuronal excitability of DRG through modulation of voltage-gated Na+ channels mediated by microtubule depolymerization, indicating that FGF13 stabilizes microtubules to modulate sodium channel function in DRG neurons and modulate inflammatory pain [75]. FGF13 deficiency was also found to alleviate mitochondrial dysfunction and paclitaxel-induced neuropathic pain by suppressing VASH1-dependent microtubule detyrosination and subsequently activating mitophagy by driving lysosomal trafficking [59,75]. Thus, targeting FGF13 may be a promising therapeutic strategy for CFA-induced inflammatory pain and paclitaxel-induced neuropathic pain [59,75].

HSP27: Chemotherapeutic agents such as paclitaxel can induce severe mitochondrial dysfunction by binding to β-tubulin located on the mitochondrial membrane [47,48]. This interaction triggers the opening of the mPTP, resulting in rapid depolarization, cytochrome c release, and ATP depletion. Structurally, this damage manifests as significant mitochondrial swelling and demyelination in distal axons. Interestingly, overexpression of human (h) HSP27 not only protects the cultured DRG neurons from mitochondrial dysfunctions after vincristine treatment [50], but also prevents the mitochondrial swelling in sciatic nerves and apoptosis in the DRG neurons [50]. More importantly, overexpression of HSP27 could prevent the development of mechanical and cold allodynia in paclitaxel-treated or vincristine-treated mice [50]. A study found that HSP27 (HspB1) can function as a molecular chaperone for the microtubule-associated protein tau, a critical regulator of the microtubule lattice [126]. It revealed that tau interacts with two distinct regions of HSP27: the structured alpha-crystallin domain (ACD) and the disordered N-terminal region (NTR). And only the interactions involving the NTR result in productive chaperone activity, which effectively prevents the pathological aggregation of tau. Mechanistically, HSP27 activity is regulated by an intramolecular interaction where the NTR is normally sequestered within a binding groove on the ACD. The release of the NTR from this groove is the key step that enhances chaperone function toward tau. By preventing tau aggregation, HSP27 supports the ability of tau to maintain the stability and integrity of the microtubule lattice [127], suggesting a mechanism of HSP27 that defends against cytoskeletal dysfunction and neurodegeneration. However, whether HSP27 prevents mitochondrial dysfunctions or the development of mechanical and cold allodynia by regulating tau and microtubules needs to be further explored.

### 5.3. Targeting the Motor Proteins

The efficacy of microtubule-targeting therapies ultimately depends on the function of motor proteins (kinesin and dynein). As introduced in Section 2, transport efficiency can be decoupled from microtubule stability under pathological conditions [128,129]. Structural disruption of microtubule dynamics is a well-characterized feature of chronic pain; however, deficits in microtubule-based transport can occur independently of detectable changes in polymer mass or stability [128]. This decoupling suggests that the stalling of cargo may not necessarily reflect a collapse of the track itself but rather a functional impairment of the machinery moving along it [129]. Consequently, directly improving cargo transport represents a therapeutic strategy that is mechanistically distinct from microtubule stabilization or acetylation manipulation and may be preferable in contexts where global modulation of microtubule dynamics is undesirable.

Kinesins are microtubule-dependent molecular motors primarily responsible for the anterograde transport (from the cell body to the synapse) of essential cargo, including mitochondria, ion channels, synaptic vesicle precursors, and neurotrophic factors [130]. In chronic pain states, deficits in kinesin-driven transport are a critical pathological mechanism leading to neuronal dysfunction and hyperexcitability [131]. Notably, kinesins have selectivity in transporting cargos [132]. In the smart motor model, kinesin selectivity is conferred by a preference of the kinesin motor domain for specific subsets of microtubules. And in the cargo steering model, kinesin selectivity is modulated by the vesicular cargo to which the motor is bound. For example, KIF17 binds to a protein complex that contains mLin-10 to transport NR2B, contributing to the development of bone cancer pain in the spinal cord [81]. And KIF17-mediated trafficking of transient receptor potential cation channel subfamily M member-3 (TRPM3) is required for the neuronal response to heat stimuli [133]. KIF13B, phosphorylated by CDK5, promotes the motor–cargo interaction and increases the anterograde transport of TRPV1, contributing to the development and possibly the maintenance of heat hyperalgesia in CFA-induced inflammatory pain [134]. KIF5B increases the membrane localizations of Nav1.7 and Nav1.8 accumulation and neuronal excitability in DRG neurons [135,136]. And mitochondrial export from soma to neurites is mainly mediated by KIF5A [137,138].

The kinesin superfamily exhibits striking cargo specificity. This specificity implies that broad, non-selective modulation of kinesins may disrupt essential transport pathways, whereas targeted strategies could correct deficits without globally perturbing transport. Collectively, these insights highlight the therapeutic promise of selectively restoring or modulating specific kinesin-driven transport routes as a means to normalize neuronal function and alleviate chronic pain.

While kinesins dominate anterograde transport, retrograde trafficking is equally essential for returning neurotrophic signals, signaling endosomes, and damaged organelles to the soma. Dynein dysfunction has been implicated in retrograde NGF signaling deficits in diabetic neuropathy [69]. And microtubule-associated retrograde transport of cargo could participate in synapse-to-nucleus communication in inflammatory pain [82]. However, direct therapeutic modulation remains largely unexplored in the pain field. Given the more limited isoform diversity of dynein compared with the kinesin superfamily, pharmacological targeting of dynein-dependent transport carries a higher risk of systemic disruption but could, in principle, exert broader translational effects.

In conclusion, the therapeutic modulation of microtubules in chronic pain necessitates a perspective that extends beyond the simple binary of polymerization and depolymerization. The pathophysiological impact of the microtubule cytoskeleton is equally mediated by post-translational modifications, the interplay with microtubule-associated proteins, and the efficiency of axonal transport. Therefore, future interventions must move toward precision to target the precise microtubule dysfunction underlying the pathology.

## 6. Limitations

Despite the compelling preclinical evidence linking microtubule dynamics to chronic pain, several critical limitations must be acknowledged to guide future research. First, a significant translational gap exists, as the vast majority of mechanistic insights derive from rodent models and cultured neurons. There is currently no direct validation of microtubule pathology in human nociceptors, nor have microtubule-targeting agents been evaluated in clinical pain trials. Second, the causal directionality of microtubule dysfunction varies considerably across pain etiologies. While chemotherapeutic agents provide a clear upstream insult, in conditions like diabetic or inflammatory pain, cytoskeletal alterations may represent downstream consequences of metabolic stress or inflammation rather than primary drivers. Disentangling cause from consequence is essential for identifying viable therapeutic windows. Finally, the therapeutic targeting of fundamental cytoskeletal components, such as tubulin, motor proteins, and microtubule-associated proteins, carries inherent risks. Because these regulators are indispensable for basic neuronal physiology and maintenance, systemic pharmacological modulation poses a substantial threat of off-target effects. Future success will therefore depend on developing strategies that achieve spatial selectivity or temporal control to alleviate pain without compromising overall neuronal integrity. Additionally, the biomarker and precision-medicine framework proposed in our concluding section remains entirely speculative. Concepts such as “microtubule phenotyping” of patient neurons, “tubulin code defect” profiling, and exosomal tubulin analysis are conceptually attractive but currently lack any empirical foundation in the pain field. Their realization would require methodological advances that do not yet exist and should be interpreted as long-term research directions rather than imminent clinical tools.

## 7. Conclusions

Chronic pain is not merely a chemical imbalance but a mechanical and logistical failure of the neuron. Future analgesic development must move beyond ion channel blockers and focus on restoring the cytoskeletal infrastructure and transport logistics of the nociceptor. We propose that achieving this requires moving toward a precision medicine paradigm. Rather than simply applying stabilizers or destabilizers in a binary manner, treatment strategies will be tailored to the disruption of microtubule stability, dysfunction of microtubule-associated protein, or cargo transport based on microtubules present in the patient’s neurons. While this framework requires substantial clinical validation, shifting the therapeutic focus from blocking electrical signals to restoring the structural and logistical integrity of the neuron via microtubule modulation holds the promise of not just alleviating symptoms but also modifying the underlying disease process of chronic pain.

## Figures and Tables

**Table 1:** The regulatory proteins that govern microtubule dynamics and transport.

Target	Model	Distribution	Expression	Mechanism	References
HDAC6	Cancer-induced bone pain	Spinal cord of rats	↑	Promotes NLRP3 inflammasome activity, inflammatory process	[102]
	CFA-induced inflammatory pain	Spinal cord of mice	↑	Promotes NLRP3 inflammasome activity, inflammatory process, astrocytic activation	[103]
	Spinal nerve ligation (SNL)-induced neuropathic pain	Spinal dorsal horn of rats	↑	Actives MyD88/NF-κB and MyD88/ERK signaling	[104]
	Chronic constriction injury (CCI)-induced neuropathic pain	Spinal cord of mice	↑	Promotes neuron activation, pyroptosis and neuroinflammatory responses	[105]
	SNI-induced neuropathic pain	Spinal dorsal horn of mice	↑	Promotes HDAC6/STAT3/CCL7 signaling axis	[106]
	Cisplatin-induced neuropathic pain	DRG neurons of mice	↑	Inhibits mitochondrial transport	[107]
	Arthritis	SW1353 cell	↑	Promotes pro-inflammatory signaling and metalloproteinase gene expression by NF-κB signaling	[108]
	Cisplatin-induced neuropathic pain	Advillin^+^ neurons of spinal cord of mice	↑	Decreases IL-10 production and IL-10 signaling and neuronal HDAC6 inhibition to restore axonal mitochondrial health	[109]
	Vincristine-induced neuropathic pain	DRG neurons of mice	↑	Disrupt axonal transport of mitochondria	[45]
	Carrageenan-induced inflammatory pain, paclitaxel-induced neuropathic pain	BV2 cells	↑	Promotes inflammatory process	[110]
	Cisplatin-induced neuropathic pain	DRG neurons of mice	↑	IENFs and mitochondrial bioenergetic deficits	[111]
Nogo-A	SNI-induced neuropathic pain/CFA-induced inflammatory pain	DRG neurons of rats	↑	Promotes microtubule polymerization and membrane localization of TRPV1	[86,87]
FGF13	CFA-induced inflammatory pain	DRG neurons of mice	↑	Promotes microtubule polymerization and Na^+^ channel activation	[59,75]
	Paclitaxel-induced neuropathic pain	DRG neurons of mice	↑	Promotes VASH1-dependent microtubule detyrosination and subsequently prevents mitophagy by disrupting lysosomal trafficking	[59,75]
HSP27	Chemotherapy-induced peripheral neuropathy	DRG neurons of mice	-	Prevents degeneration of afferent nerve fibers, demyelination, mitochondrial swelling, apoptosis, and restored sensory nerve action potential	[50]
CRMP2	SNI-induced neuropathic pain	Spinal dorsal horn, DRG neurons of rats	↑	Interacts with and facilitates the forward trafficking of N-type voltage-gated calcium channels (Cav2.2) and sodium channels (Nav1.7) to the membrane	[112,113]

Abbreviations: HDAC6, Histone deacetylase 6; CFA, complete Freund’s adjuvant; SNL, spinal nerve ligation; MyD88, Myeloid Differentiation Primary Response 88; NF-κB, Nuclear Factor kappa-light-chain-enhancer of activated B cells; ERK, Extracellular Signal-Regulated Kinase; NLRP3, NOD-like receptor pyrin domain containing 3; CCI, chronic constriction injury; SNI, spared nerve injury; STAT3, Signal Transducer and Activator of Transcription 3; CCL7, C-C Motif Chemokine Ligand 7; IL-10, Interleukin-10; DRG, dorsal root ganglion; IENFs, intraepidermal nerve fibers; TRPV1, transient receptor potential vanilloid subfamily member 1; FGF13, fibroblast growth factor 13; VASH1, vasohibin 1; HSP27, Heat Shock Protein 27; CRMP2, Collapsin Response Mediator Protein 2.

**Table 2:** The selective HDAC6 inhibitor applying to chronic pain.

Inhibitor	Model	Site of Action	Efficiency	Mechanism	References
TSA	Cancer-induced bone pain	Spinal cord of rats	Alleviates spontaneous pain and mechanical hyperalgesia and recovers motor coordination	Decreases spinal inflammatory cytokine production, suppresses spinal astrocytes activation, and reduces NLRP3 inflammasome activity	[102]
TSA	Arthritis	SW1353 cell	-	Reduces matrix metalloproteinase (MMP) expression, blocks cytokine-induced signaling and reduces the cartilage degradation by NF-κB signaling	[108]
ACY1215	SNL-induced neuropathic pain	Spinal dorsal horn of rats	Alleviates mechanical allodynia	Reduces MyD88/NF-κB and MyD88/ERK	[104]
ACY1215	CCI-induced neuropathic pain	Spinal cord of mice	Alleviates mechanical allodynia, but not thermal hyperalgesia	Inhibits neuron activation and suppresses CCI-induced pyroptosis and neuroinflammatory responses	[105]
ACY1215	SNI-induced neuropathic pain	Spinal dorsal horn of mice	Alleviates mechanical hypersensitivity	Suppresses HDAC6/STAT3/CCL7 signaling axis	[106]
ACY1215	Cisplatin-induced neuropathic pain	DRG neurons of mice	Reverses mechanical allodynia, spontaneous pain, and numbness	Increases mitochondrial transport	[107]
ACY1083	Cisplatin-induced neuropathic pain	Advillin^+^ neurons of spinal cord of mice	Alleviates mechanical hypersensitivity	Promotes IL-10 production and IL-10 signaling and neuronal HDAC6 inhibition to restore axonal mitochondrial health	[109]
ACY-738, ACY-257	SNI-induced neuropathic pain, CFA-induced neuropathic pain	mice	Attenuates mechanical allodynia but not thermal hyperalgesia	Peripheral inflammation	[118]
ACY-738	Vincristine-induced neuropathic pain	DRG neurons of mice	Alleviates mechanical hypersensitivity	Reverses axonal transport of mitochondria	[45]
compound 13, compound 36	Carrageenan-induced inflammatory pain, paclitaxel-induced neuropathic pain	BV-2 cells	Alleviates mechanical allodynia	Inhibits inflammatory process	[110]
CKD-011	Chemotherapy (oxaliplatin, paclitaxel, and cisplatin)-induced neuropathic pain	Sciatic nerve of rats	Alleviates mechanical hypersensitivity	None	[120]
ITF6464, ITF6475	Cisplatin-induced neuropathic pain	DRG of mice	Alleviates mechanical hypersensitivity	Protects the damaged IENFs	[119]

Abbreviations: TSA, tubastatin A; NLRP3, NOD-like receptor pyrin domain containing 3; MMP, matrix metalloproteinase; MyD88, Myeloid Differentiation Primary Response 88; NF-κB, Nuclear Factor kappa-light-chain-enhancer of activated B cells; ERK, Extracellular Signal-Regulated Kinase; SNL, spinal nerve ligation; CCI, chronic constriction injury; SNI, spared nerve injury; HDAC6, Histone deacetylase 6; STAT3, Signal Transducer and Activator of Transcription 3; CCL7, C-C Motif Chemokine Ligand 7; DRG, dorsal root ganglion; IL-10, Interleukin-10; CFA, complete Freund’s adjuvant; IENFs, intraepidermal nerve fibers.

## Data Availability

No new data were created or analyzed in this study. Data sharing is not applicable to this article.

## Abbreviations

MT, microtubule; HDAC6, Histone deacetylase 6; CRMP2, Collapsin Response Mediator Protein 2; HSP27, Heat Shock Protein 27; PTMs, post-translational modifications; MTs, microtubules; MTOCs, microtubule-organizing centers; GTP, Guanosine Triphosphate; MAPs, microtubule-associated proteins; ATP, Adenosine Triphosphate; ER-PM, endoplasmic reticulum-plasma membrane; CIPN, chemotherapy-induced peripheral neuropathy; IENFs, intraepidermal nerve fibers; VDAC, Voltage-Dependent Anion Channel; MNK, Mitogen-Activated Protein Kinase-Interacting Kinase; eIF4E, Eukaryotic Translation Initiation Factor 4E; mPTP, mitochondrial permeability transition pore; DRGs, dorsal root ganglions; DRG, dorsal root ganglion; FGF13, fibroblast growth factor 13; VASH1, vasohibin 1; DPN, diabetic peripheral neuropathy; AGEs, advanced glycation end-products; RAGE, advanced glycation end-products receptor; GSK-3β, glycogen synthase kinase-3β; TRPV4, transient receptor potential cation channel subfamily V member 4; NGF, Nerve Growth Factor; CFA, complete Freund’s adjuvant; NMDAR, N-methyl-D-aspartate receptor; CREB, cAMP response element-binding protein; BDNF, Brain-Derived Neurotrophic Factor; VR1, vanilloid receptor subtype 1; SCI, spinal cord injury; SNI, spared nerve injury; TPPP3, polymerization-promoting protein 3; SP, substance P; NLRP3, NOD-like receptor pyrin domain containing 3; IL-6, Interleukin-6; CCD, chronic compression of the DRG; NAP, Davunetide; TRPV1, transient receptor potential vanilloid subfamily member 1; TSA, tubastatin A; CDK5, Cyclin-Dependent Kinase 5; SNL, spinal nerve ligation; CCI, chronic constriction injury; MMP, matrix metalloproteinase; ACD, alpha-crystallin domain; NTR, N-terminal region; TRPM3, transient receptor potential cation channel subfamily M member-3.
